# ASO Author Reflection: Is There a Role for Cardiopulmonary Exercise Testing Before Esophagectomy?

**DOI:** 10.1245/s10434-021-10183-y

**Published:** 2021-05-27

**Authors:** Jakub Chmelo, Alexander W. Phillips

**Affiliations:** Northern Oesophagogastric Unit, Royal Victoria Infirmary, Newcastle upon Tyne, UK

## Past

Esophagectomy is recognized as a high-risk procedure associated with significant morbidity and mortality. Prognosis is most commonly associated with pathological stage of the disease.^1^ In some centers cardiopulmonary exercise testing (CPET) has been used to try and predict patients at high risk of perioperative complications with a correlation that poorer cardiopulmonary fitness is associated with a greater chance of complications.^2^ CPET also may identify patients with undiagnosed chronic conditions that may impact on long-term outcomes. Hitherto, this has not been explored in patients undergoing esophagectomy.

## Present

The present study reveals that variable of ventilatory inefficiency (VE/VCO_2_) derived from preoperative CPET is independently predictive of overall survival after transthoracic esophagectomy for cancer.^3^ Whilst the receiver operation curve (ROC) analysis did not demonstrate a cut-off value that was sensitive and specific as high risk, the stratification of groups does demonstrate a stepwise impact on long-term survival. Importantly, the findings from this study demonstrate a further factor that may be important to consider during the multidisciplinary process. It adds to the argument for the use of CPET in preoptimization of patients, as some conditions associated with elevated VE/VCO_2_ can be addressed in preoperative period.

## Future

CPET before esophagectomy is not able to identify, with high sensitivity or specificity, patients with poor postoperative outcomes.^4^ However, its important role lies in aiding to the shared decision-making process in regard to the risk and benefit of surgery or in identifying patients who might benefit from preoperative optimization. Further research is required into this area to determine if CPET is a useful tool in prehabilitation and can help to guide patient optimization before surgery.

## References

[1] Davies AR, Gossage JA, Zylstra J (2014). Tumor stage after neoadjuvant chemotherapy determines survival after surgery for adenocarcinoma of the esophagus and esophagogastric junction. J Clin Oncol..

[2] Sheill G, Reynolds S, O'Neill L, et al. Cardiopulmonary exercise testing in oesophagogastric surgery: a systematic review. *J Gastrointest Surg*. 2020;24(11):2667–78. 10.1007/s11605-020-04696-2 32632727

[3] Chmelo J, Khaw RA, Sinclair R, Navidi M, Phillips AW. Does cardiopulmonary testing help predict long-term survival after esophagectomy? *Ann Surg Oncol*. 2021. 10.1245/s10434-021-10136-5. 10.1245/s10434-021-10136-5 PMC8519940 34041625

[4] Stubbs DJ, Grimes LA, Ercole A (2020). Performance of cardiopulmonary exercise testing for the prediction of post-operative complications in non cardiopulmonary surgery: a systematic review. PLoS One..

